# Rassi Score for Secondary Prevention of Sudden Cardiac Death in Chagas Heart Disease

**DOI:** 10.4269/ajtmh.26-0215

**Published:** 2026-04-16

**Authors:** Henrique Horta Veloso, Alejandro Marcel Hasslocher-Moreno, Roberto Magalhães Saraiva

**Affiliations:** Evandro Chagas National Institute of Infectious Diseases, Osvaldo Cruz Foundation, Rio de Janeiro, Brazil, E-mails: henrique.veloso@ini.fiocruz.br, alejandro.hasslocher@ini.fiocruz.br, roberto.saraiva@ini.fiocruz.br

Dear Editor,

In the interesting case report of Caplan et al.,^1^ a Latin-American patient attended to in an emergency department in the northeastern United States presented with sustained ventricular tachycardia (VT), initially controlled with intravenous amiodarone. Subsequent transthoracic echocardiography revealed a nonischemic cardiomyopathy with posterior ventricular wall hypokinesis and left ventricular (LV) aneurysm. Cardiac magnetic resonance imaging showed inferolateral/apical scarring, a finding suggestive of remote myocarditis, and a LV ejection fraction of 38%. Two serological tests confirmed the diagnosis of Chagas cardiomyopathy. Based on this information, the authors stated that “given an elevated sudden cardiac death (SCD) risk (Rassi score 15), a single-chamber implantable cardioverter-defibrillator (ICD) was implanted, followed by a successful VT ablation”.^1^ In our opinion, the use of the Rassi score to define the use of an ICD in this case was not appropriate.

The risk Rassi score for predicting SCD in Chagas cardiomyopathy was developed and validated for the primary prevention setting.^2^ In that Rassi study,^2^ patients with Chagas cardiomyopathy were enrolled, but those with sustained VT or ventricular fibrillation were excluded because of a high risk of death. So, the case reported with sustained VT, consequently considered in the secondary prevention setting, would not have been included in the study.

As the authors stated, “although United States guidelines lack Chagas-specific ICD recommendations, secondary prevention with ICD after VT and structural heart disease is standard per American College of Cardiology criteria”. According to the 2017 AHA/ACC/HRS Guideline,^3^ an ICD is recommended for this patient since he had a nonischemic cardiomyopathy and experienced a stable, sustained VT, not due to reversible causes (Class of Recommendation I). Current guidelines on the diagnosis and treatment of patients with cardiomyopathy of Chagas disease also recommend an ICD for this patient since he presented with stable sustained VT and had an LV ejection fraction ≤40%.^4^ It is important to note that a Rassi score was not considered to reach this recommendation.

Two recent studies investigated the role of the Rassi score for secondary prevention of SCD in Chagas disease.^5^^,^^6^ Campos et al.^5^ observed that in patients with Chagas cardiomyopathy treated with an ICD, the vast majority (90%) of whom received the device for secondary prevention, high defibrillation threshold testing was related to high Rassi scores. Pereira et al.^6^ observed that in patients with Chagas cardiomyopathy treated with an ICD, the majority (74%) of whom received the device for secondary prevention, an intermediate Rassi score was associated with a high incidence of appropriate activation of the device.

In summary, we believe that the Rassi score is an important tool to stratify patients with Chagas cardiomyopathy in the primary prevention of SCD. However, it should not yet be used for secondary prevention. Further studies may investigate its utility in this context.
